# Child-rearing Support Provided by Public Health Nurses to People with Mental Illness: Qualitative Descriptive Study

**DOI:** 10.2174/1874434601812010162

**Published:** 2018-07-31

**Authors:** Masako Kageyama, Keiko Yokoyama

**Affiliations:** 1 Department of Health Promotion Science, Osaka University Graduate School of Medicine, Yamadaoka, Suita, Osaka, Japan; 2 Department of Nursing, Faculty of Health Sciences, Saitama Prefectural University, Saitama, Japan

**Keywords:** Mental illness, Child-rearing, Parenting, Public health nurses, Maternal health, Child abuse

## Abstract

**Background:**

The growing rates of deinstitutionalization in Japan have resulted in an increase in the number of children being raised by parents with mental illness. Given this situation, public health nurses working for local governments play an important role.

**Objective:**

The purpose of this qualitative descriptive study was to describe the child-rearing support provided by public health nurses to parents with mental illness.

**Methods:**

Seven nurses identified 28 cases of parents with mental illness. Descriptions of the goals and details of the appropriate nursing support were extracted from transcripts, coded, and categorized.

**Results:**

Parents with mental illness diagnosed with addiction and personality disorders were more difficult to support than those diagnosed with mood disorders or schizophrenia. Public health nurses supported parents with mental illness with the aim of achieving goals such as “building continuous consultative relationships,” “ensuring living conditions had a minimum level of safety and comfort,” “parents playing their roles,” “parents and children living together in the community,” and “fostering children’s growth.” While they provided support by “assessing their relationships with parents,” “building consultative relationships with parents,” “assessing parents’ illnesses/disorders and supporting,” “assessing child-rearing abilities and supporting,” and “cooperating with related agencies,” they tended to focus on the growth of the children rather than the recovery of parents.

**Conclusion:**

Consultative relationships were the beginning of support, as well as the most important and difficult skill. Public health nurses need to provide support for the recovery of parents with mental illness and learn about personality disorders and addiction.

## 1 INTRODUCTION

In Western countries, deinstitutionalization and community-based rehabilitation and support programs have increased the likelihood of persons with Mental Illness (MI) becoming parents and raising children [1]. In the U.S., 68% of women and 54.5% of men with a non-substance MI are slightly more likely to be parents than those without MI (62.4% and 52.9% respectively) [2]. Women and men with MI are at least as likely as those without MI to become parents [1, 2]. A national survey in Japan showed that 25% of persons with MI were living with spouses and 16.7% of persons with MI were living with their children [3]. Another survey showed that 66% of persons with MI wanted to get married [4]. In the future, more persons with MI are expected to get married and have children, making parenting an increasingly important issue in the acceleration of deinstitutionalization in Japan. However, there are no specific support programs or guidelines for Parents with MI (PMI) in Japan, making it necessary to develop the relevant support services and skills.

However, children of PMI are at significantly greater risk for multiple psychosocial problems. Rates of child psychiatric diagnosis among offspring range from 30% to 50%, compared to an estimated rate of 20% among the general child population [2]. Moreover, in Japan, children of PMI who live in child welfare facilities are half as likely to be abused by their parents [5]. Evidence from several reports in Japan indicates that a half to one-third of child abuse is perpetrated by PMI [6]. There is strong evidence that PMI are more likely to abuse their children than parents without MI [7]. Indeed, children living with PMI are at greater risk of adverse outcomes than children not living with PMI [7].

Research on PMI has primarily focused on the adverse effects on children [8]. Little research has been produced on developing parenting programs or describing the support skills of practitioners in Japan. By law, public health nurses (PHNs) working in local government have an important role to play in the care of mothers and children. However, PHNs are likely to experience difficulties in providing individual consultations for residents with mental health issues [9]. Their difficulties increase even further when providing support for PMI, who are at a high risk of perpetrating child abuse compared to parents without MI [10]. Regarding research about PHNs’ support skills for PMI, there has been secondary qualitative analysis based on 13 published case reports. However, this qualitative data is insufficient and, moreover, most of these case reports were conducted over 20 years ago [11]. Delineating PHNs’ support skills for PMI is important in the prevention of child abuse as well. In other countries as well as Japan, many practitioners do not have the required training to work with PMI and their children. Parenting was rated to be the greatest learning need across all practitioners [12].

In order to prepare for the future increase in PMI, this study aimed to elucidate child-rearing support provided by PHNs for persons with MI.

## 2 MATERIALS AND METHODS

### 2.1 Research Design

A clear-cut description of the as-yet-unexamined aspect of parenting support for persons with MI. We, therefore, performed a qualitative descriptive study [13].

### 2.2 Interview Participants

Those interviewed included PHNs currently working at the local government maternal department who had experience of 10 years or more in providing support for PMI. Three local governments were selected. The researcher contacted the manager in charge of research for PHNs in each government. Seven PHNs met the inclusion criteria. First, we sent research documents to them and explained the study telephonically. Subsequently, the study was explained in person and the interview was conducted.

### 2.3 Data Collection

The interviews were semi-structured and conducted using an interview guide. Participants were asked to select a total of four cases—two cases they judged to be examples of successful support provision and two cases that were judged unsuccessful. We thought if we directly asked participants their goals, they would mention the healthy growth of children, which is their role as per the law. Accordingly, we asked participants to choose two cases each that they judged to be most successful and most unsuccessful. By getting insight into the criteria they used for judging support to be successful or otherwise, we hoped to gain an understanding of their support goals. Moreover, we expected that this method would provide rich data about support skills.

Participants were asked for the following: (1) case summaries, (2) reasons for their judgment of support as successful or unsuccessful, and (3) the content of their support, including assessment and behaviors. The interviews lasted one to two hours each. The length of time for each case was 15±5 minutes (range: 7–24). Interview data were recorded and transcribed.

### 2.4 Data Analysis

First, summaries of the 28 cases of support provided by the PHNs were organized. Second, interview data were analyzed and sorted into qualitative descriptions. Descriptions of the goals or content of support were extracted from the transcripts and segmented by meaning. Each segment was coded and labeled according to the following two research questions: “What were the goals of your support?” and “How did you provide support?” Data were categorized and subcategorized based on the similarity of codes.

To ensure the trustworthiness and rigor of the findings [14], the first author analyzed all data and the second author confirmed the first author’s interpretation through a discussion. The study’s rigor was increased by requesting the interviewed participants to endorse the results. Five participants replied that they agreed with the results and that they would be valuable in practice.

### 2.5 Ethical Considerations

The objectives of the study, voluntary basis of participation, confidentiality, and autonomy were explained to participants orally and in writing using the Participant Information Sheet. Written consent was obtained before each interview. This study was approved by the Research Ethics Committee, Faculty of Medicine, Osaka University (16283).

## 3 RESULTS

### 3.1 Demographic Characteristics of Participants

The seven PHNs were women with an average age of 44±7.2 (range: 35–55). It was reported that six PHNs had provided support in 20 or more PMI cases each, while the remaining PHN reported having provided support in under 20 cases. All participants had provided support for pregnant women and potentially abused children, visiting services for babies and toddlers, and group sessions for parents who had abused their children.

### 3.2 Characteristics of Cases

Of 28 cases, there were 20 where only the mother suffered from MI, no cases where only the father suffered from MI, and eight cases where both the mother and father suffered from MI. The diagnoses of each case are shown below in Table **1**. Diagnoses were duplicated in seven cases. Support in cases with addiction and personality disorders was likely to be unsuccessful.

### 3.3 The goals of support as Perceived by PHNs

Support was provided with the aim of achieving goals such as “*building continuous consultative relationships*,” “*ensuring living conditions had a minimum level of safety and comfort*,” “*playing roles as parents*,” “*parents and children living together in the community*,” and “*fostering children’s growth*.” Each of these categories is supported by quotes from interview transcripts (case ID).

#### 3.3.1 Building Continuous Consultative Relationships

If they could build continuous consultative relationships with parents, PHNs judged their support to be successful.

Successfully supported: “We provided support as soon as possible after receiving an SOS from the parents. Through the support, we built a good consultative relationship through which we could help the mother deal with the psychiatric hospital smoothly” (No. 24).

Unsuccessfully supported: “The mother insisted that we should not visit the home. However, as I was deeply concerned about the child, I visited them. This resulted in the mother consistently refusing support from PHNs” (No. 27).

#### 3.3.2 Ensuring Living Conditions Had a Minimum Level of Safety and Comfort

Based on whether the family’s living conditions had a minimum level of safety and comfort, including keeping the parents in a stable medical condition, avoiding crises, or ensuring children’s safety, the PHNs judged their support to be successful.

Successfully supported: “We collaborated closely with other support agencies. Therefore, we were aware of situations before they turned into crises” (No. 13).

Unsuccessfully supported: “The father perpetrated violence against the mother. We regret that the mother got pregnant a second time. We are unsure if they can raise the baby” (No. 19).

#### 3.3.3 Parents Playing Their Roles

Based on whether they played their roles as parents, which meant that they tried to parent even if their attempts were deficient, or that they called for help when they could not cope with issues, PHNs judged the support they provided to PMI to be successful.

Successfully supported: “I thought the mother made her best efforts in child-rearing” (No. 21).

Unsuccessfully supported: “The mother could not respond to him well and saw him with a cold eye” (No. 23).

#### 3.3.4 Parents and Children Living Together in the Community

Based on whether the parents and children lived together in the community, PHNs judged their support to be successful.

Successfully supported: “The child goes to a daycare center. I judged our support to be successful because they live together at home, although the family sometimes confronts difficult issues” (No. 17).

Unsuccessfully supported. “I judged our support to be unsuccessful because they lived separately as a result of our failure to achieve family reunification” (No. 15).

#### 3.3.5 Fostering Children’s Growth

Based on whether the children grew well, which means they were born healthy and grew up physically and psychologically healthy, PHNs judged the support they provided to PMI to be successful. Support was judged unsuccessful when the children grew up unhealthy or, like their parents, became part of a generational chain of abuse.

Successfully supported: “The son liked to stay home and refused to go to school. However, he said that he had recently started enjoying school and playing soccer” (No. 10).

Unsuccessfully supported: “I am worried that the children might grow up to be small versions of their parents” (No. 23).

### 3.4 Support Skills

Support skills included the following: “*assessing their relationships with parents*,” “*building consultative relationships with parents*,” “assessing illnesses/disorders and supporting,” “assessing child-rearing abilities and supporting,” and “cooperating with related agencies.”

The categories of “*assessing their relationships with parents”* and *“building consultative relationships with parents”* include most data related to the support skills used by the PHNs; essentially, these categories are concerned with the most important skills related to supporting PMI.

#### 3.4.1 Assessing Their Relationships with Parents

Nurses assessed their relationships with parents by the reactions of the parents or those close to them, including statements like “I am relieved when I see you (PHN)” (No. 25), expressions like “gloomy faces” (No. 3), patterns of contact like “talking for several hours when the mother wants” (No. 6), and communication style used like “complainer everywhere” (No. 23). Building consultative relationships with PMI was deemed difficult when their interactions consisted of *persistent superficial talk*, when there were *consultations with staff other than the designated staff*, when *the relationship was cut off*, when *contact was self-serving*, when there was *instability*, and *the presence of communication impairment*.

*Persistent superficial talk*: “The mother did not object to my home visits. However, I felt our relationship was not getting deep because she only talked about superficial issues whenever I visited” (No. 3).

*Consultations with staff other than the designated staff*: “The mother called staff from other agencies when she wanted a consultation” (No. 6).

*Relationship is cut off because of discomfort:* “The mother reproached PHNs she did not like and cut off relationships with them” (No. 23).

*Self-serving contact:* “The mother cancels appointments at the last minute or does not come out when I knock on the door. She contacts us at her convenience and does not care for others” (No. 19).

*Instability:* “The mother told various people different stories. While she told one person she was a victim of domestic violence, she told another person that she had a good relationship with her husband” (No. 23).

*The presence of communication impairment:* “The mother was obsessive. If I made a suggestion she would go into a bad mood ” (No. 28).

#### 3.4.2 Building Consultative Relationships with Parents

Consultative relationships were built with parents who had difficulties in forming relationships with others by *listening to the parents well*; *learning about past relationship patterns*; *continuous support*; *focusing on healthy aspects, not unhealthy ones*; and *providing support along with a person the parent relied on*.

*Listening to the parents well:* “What I did was to listen to the mother very well” (No. 25).

*Learning about past relationship patterns*: “Past consultation records showed me that the mother had shut out previous PHNs. Therefore, I was careful about timing if I had to make suggestions” (No. 6).

*Continuous support:* “I have been supporting the mother continuously for three years” (No. 18).

*Focusing on healthy aspects, not unhealthy ones:* “I was consulted about the mother’s mental condition by her sister. However, I told the mother that my home visit was for her baby” (No. 1).

*Providing support along with a person the parent relied on:* “A midwife had provided continuous support to the mother. Therefore, when I began to support her, I sometimes took the midwife along on my visits” (No. 26).

However, PHNs reported many failures in forming consultative relationships, which they believed were owing to the following reasons: *insufficient understanding of the person’s history*, *insufficient acceptance when the parent opened up, inability to make direct contact*, *unwanted how to support*, and *parents’ dissatisfaction with the scope of support*.

*Insufficient understanding of the person’s history:* “I should have paid more attention to the father’s history. Had I done that, I might have been able to counsel them on family planning and prevent the mother’s current pregnancy” (No. 19).

*Insufficient acceptance when the parent opened up:* “The mother confessed to me about her experience of sexual abuse in early childhood. I was not expecting it, so I could not accept her open disclosure” (No. 3).

*Inability to make direct contact:* “The mother was busy with work, so I could not contact her at the right time” (No. 4).

*Unwanted how to support:* “The mother wanted to receive the kind of special treatment the previous PHN gave her. I supported her in the usual manner and she refused my support” (No. 7).

*Parents’ dissatisfaction with the scope of support:* “The mother was emotionally addicted to people. She called us every day. We offered in-person counseling once a month. However, she was not satisfied with this frequency and refused our support” (No. 15).

#### 3.4.3 Assessing Illness Disorders and Supporting

The PHNs evaluated the need for support by *assessment of the parents’ mental illness*, and *assessment of abilities of child-rearing related to symptoms*. Based on the assessment, PHNs *supported families in cooperation with psychiatrists*.

It is possible for diagnoses of MI to change; therefore, the PHNs performed their own assessments, for instance, “I believed the diagnosis was wrong” (No. 21). However, they sometimes misdiagnosed PMI or misunderstood their symptoms, for example, “I believed she had a developmental disorder. Therefore, my support to her was inadequate” (No. 15). Their misunderstanding of MI impeded their capacity to support the patients adequately.

*Assessment of parents’ MI:* “The mother insisted her husband committed domestic violence and it was difficult to raise the child. Therefore, I thought the child had a developmental disorder. However, the child did not have developmental issues and the husband did not commit any violence. All of this was a delusion by the mother. I regret not having contacted the husband” (No. 16).

If the burden of child-rearing is substantial, the symptoms of PMI can worsen, affecting their capacity to care for children. Accordingly, PHNs assessed their symptoms and parenting capacity.

*Assessment of abilities of child-rearing related to symptoms:* “The mother did not sleep at night, cook in the morning, and gave milk to baby” (No. 25).

By contacting the parents’ psychiatrists, PHNs provided support. However, when PHNs were unable to cooperate with the psychiatrists, there could be bad outcomes for parents.

*Supporting the family by cooperating with the psychiatrist:* “The mother repeatedly overdosed on medication. She passed away. I could not consult with the psychiatrist well” (No. 11).

#### 3.4.4 Assessing Child-rearing Abilities and Supporting

Child-rearing capacity was assessed in terms of categories such as *affection for the child*, for example, “did not react if the baby cried or laughed” (No. 28); *child-rearing skills*, for example, “asked me whether it was all right to give the baby rice cakes” (No. 10); and *basic life stability*, for example, “gathered a lot of trash at home because of his collectomania” (No. 28).

*Child-rearing Skills:* “The mother gave the baby milk; however, she could not prepare it correctly. The amount of milk and the manner of preparation were wrong” (No. 26).

Child-rearing support was provided by *giving consultations each time* like “She was worried about little things, so I taught her each time” (No.13); *gaining the cooperation of relatives*, for example, “I visited the grandparents’ home many times and asked for assistance in child-rearing” (No. 17); and *using support services fully*, for example, “I made the parents use social resources as much as possible” (No. 14).

Giving consultations each time: “We told the mother to come to us whenever she is irritated at her baby.” (No. 21).

#### 3.4.5 Cooperating with Related Agencies

To support PMI, PHNs needed to collaborate with staff of the mental health department, child welfare agencies, psychiatrists, and other related agencies. They cooperated with related support agencies by *sharing roles*, for example, “We ask for the child consultation center’s help when the situation gets too severe” (No. 20); by *coordinating the management of support*, for example, “Many agencies are involved with the case. The management is important” (No.28); and *providing timely support*, for example, “I visited the mother soon after her SOS” (No. 22).

*Coordinating the management of support:* “I made arrangements to support the parents during pregnancy and the immediate postpartum period by using the help of caretakers, caseworkers, home visiting staff for child-rearing, planning support staff, and medical social workers” (No. 9).

## 4 DISCUSSION

### 4.1 Diagnoses of MI and Difficulties in Providing Support

In the current study, each participant reported two cases she felt had been examples of successful support and two cases of unsuccessful support. Support in cases of addiction and personality disorders was likely to be unsuccessful. A literature review revealed that the parents of abused children largely suffer from mood disorders, schizophrenia, addiction, and personality disorders [6]. In the current study, cases of addiction and personality disorders were likely to be more difficult than cases of mood disorders or schizophrenia. Previous reports have suggested that addiction [15] and personality disorders are [16] are characterized by significant difficulties in child-rearing. Moreover, these disorders basically cannot be treated with medication. These might have been the reason why PHNs were unsuccessful in supporting parents with addiction or personality disorders.

### 4.2 Goals of Support as Perceived by PHNs

Nurses supported PMI with the goals of “building continuous consultative relationships,” “ensuring that living conditions had a minimum level of safety and comfort,” “playing roles as parents,” “parents and children living together in the community,” and “fostering children’s growth.” It is important to note that the recovery of PMI, which refers to gaining and retaining hope, understanding one’s abilities and disabilities, engagement in an active life, personal autonomy, social identity, meaning and purpose in life, and a positive sense of self, was not among their goals [17]. Practitioners supporting parents and children rarely follow the goals associated with mental health recovery models [18]. However, recovery is a major goal of mental health rehabilitation and should accordingly be recognized as one in the context of PMI.

### 4.3 Skills Required to Support PMI

The current study revealed that PHNs supported PMI with the following skills: “assessing their relationships with parents,” “building consultative relationships with parents,” “assessing illnesses/disorders and supporting,” “assessing abilities of child-rearing and supporting,” and “cooperating with related agencies.” In particular, “assessing their relationships with parents” and “building consultative relationships with parents” not only marked the beginning of support but also proved the most important, and most difficult, skills. When PHNs were unsuccessful in providing support to PMI, it was because they had been unsuccessful in forming relationships with them . Another study reported that 40% of PHNs found it difficult to provide support for child abuse when the parents’ mental health was unstable and they refused their involvement [19]. The main reasons why PHN face difficulties in providing support in child abuse cases can be an inability to involve parents or parents refusing their support [20]. Ensuring that the process of providing support does not result in the person with MI cutting off the relationship was described as an important skill in another study [21]. However, studies describing these skills were focused on supporting victims of child abuse or persons with MI; they did not focus on PMI. If PMI refuse PHNs’ involvement, it becomes difficult for them to gain access to the children, which could worsen the child abuse. Even training programs for professionals have not focused on building relationships with parents [12]. The current study sheds light on how to assess relationships with PMI and how to build them further, which may be beneficial for PHNs. It is common for PHNs to be underconfident about supporting persons with MI [22, 23]. The ability to provide effective consultations concerning child abuse is related to the ability to provide effective mental health consultations in general [10]. By increasing their exposure to mental health consultations, PHNs can develop their own support skills for child abuse [24]. Therefore, PHNs are encouraged to experience and learn about support skills related to mental health issues.

### 4.4 Implications for Practice

There is limited research about supporting PMI in Japan. As PHNs experience more difficulties when providing support for PMI [10], the results of the current study can be useful in creating relevant guidelines specific to Japan. Moreover, the support skills for building relationships with PMI described in the current study can serve as useful references for mental health practitioners when supporting PMI in other countries.

It is important for PHNs to have meetings to examine cases and discuss the provision of support in difficult cases, including PMI [25]. The interviewed PHNs provided 28 cases rich in information and experiential knowledge, which will be useful in the field. If PHNs can use these cases during case examinations, it will be beneficial to them. Next, PHNs are recommended to gain experience in supporting persons with MI and attend lectures on the topic as they sometimes misunderstand diagnoses and how to provide support. It is especially recommended that PHNs learn about personality disorders and addiction.

### 4.5 Research Limitations and Future Research

Owing to the design of this study, the results cannot be generalized. In addition, the participants were from three local governments; therefore, the results may not be the same in other governments. As PHNs can face difficulties in providing support for PMI, the development of learning programs for the former will be valuable as future research.

## CONCLUSION

Seven PHNs provided 28 cases of PMI. Among the diagnoses, people suffering from addiction and personality disorders were likely to be more difficult to support than those with mood disorders or schizophrenia. The PHNs did not support the recovery of persons with MI. Building consultative relationships, which is the beginning of the process of providing support, is the most important, and most difficult, skill. It is necessary for PHNs to provide support for the recovery of PMI and learn about personality disorders and addiction.

## Figures and Tables

**Table 1:** Diagnoses of cases supported successfully or unsuccessfully.

Diagnosis (Multiple Diagnoses)	Supported Successfully	Supported Unsuccessfully	Total
Schizophrenia	6	4	10
Mood disorders	8	5	13
Addiction or personality disorders	2	7	9
Adjustment disorders	0	2	2
Panic disorders	1	0	1
Dissociative disorders	0	1	1
Total	17	19	36

Eight cases had two diagnoses.
